# PP69 Cost Calculator For Health Technology Assessments In Intensive Care Units: Application Of Machine Learning

**DOI:** 10.1017/S0266462325102870

**Published:** 2025-12-29

**Authors:** Isabella Bezerra, Gabriela Campino, Antônio Paulo Nassar Junior, Janaina Germano, Augusto Vellozo, Wadson Bezerra, Daniel Malheiro, Adriano Jose Pereira

## Abstract

**Introduction:**

Intensive care unit (ICU) hospitalization costs are relevant due to their high impact on the healthcare system. ICU admissions financed by the Brazilian government through the Unified Health System totaled USD2.260 billion in 2023. Given the magnitude of these expenditures, economic evaluations of innovations in ICU care are critical. Because of the limitations of conventional sources of cost data, this study aimed to develop a cost estimation tool for health technology assessments.

**Methods:**

Data were extracted from the IMPACTO-MR Project, a collaborative research platform coordinated by Brazilian hospitals, including Hospital Israelita Albert Einstein, partnering with the Ministry of Health and the National Health Surveillance Agency (ANVISA). Patient-level data were collected from 15 Brazilian ICUs from October 2019 to October 2024. Costs were categorized into fixed costs (doctors, nurses, physiotherapists, nursing assistants, administrative assistants, other workers, depreciation, electricity, water, telephone, internet, indirect costs, office supplies) and variable costs (drugs, materials, hemodialysis, blood transfusions, laboratory and imaging tests, medical gases). ICU costs were estimated by identifying consumption patterns using machine learning and considering clinical variables. Costs were adjusted for purchasing power parity and inflation.

**Results:**

ICU costs per patient showed substantial variability. Fixed costs were more related to the structure of the ICU, management practices, and local and regional characteristics, did not vary in the short term, and had a strong correlation with length of stay. Variable costs were more related to the patient characteristics, reflected direct consumption, and varied in the short term. Estimating costs for health technology comparisons was possible with this calculator. Machine learning can be used to identify consumption patterns in ICUs and help estimate costs while taking into account clinical variables.

**Conclusions:**

The IMPACTO-MR Project is conducting several health technology assessments in ICUs in Brazil. With this calculator, it is possible to perform cost-effectiveness analyses in ICUs when cost data are not available.

